# Macular Retinal Layer Thickness in Mild-to-Moderate Parkinson’s Disease

**DOI:** 10.3390/diagnostics16203412

**Published:** 2026-10-20

**Authors:** Michael Y. Zhu, Sejal D. Patel, Nikki Daniels, John Hsu, Rachel D’Cunha, Alex Choi, Alice Haystead, Stephanie J. Chiu, Burton Scott, Dilraj S. Grewal, Sharon Fekrat

**Affiliations:** 1 iMIND Study Group, Department of Ophthalmology, Duke University School of Medicine, Durham, NC 27705, USA; m.zhu@duke.edu (M.Y.Z.); sejal.d.patel@duke.edu (S.D.P.); nikki.daniels@duke.edu (N.D.); john.hsu@duke.edu (J.H.); rachel.dcunha@duke.edu (R.D.); alex.choi@duke.edu (A.C.); alice.haystead@duke.edu (A.H.); stephanie.chiu@duke.edu (S.J.C.); dilraj.grewal@duke.edu (D.S.G.); 2 Department of Neurology, Duke University School of Medicine, Durham, NC 27710, USA; burton.scott@duke.edu; 3 Duke University Medical Center, 2351 Erwin Road, Durham, NC 27705, USA

**Keywords:** Parkinson’s disease, optical coherence tomography, retinal layer thickness, neurodegeneration, biomarkers, macula

## Abstract

**Background/Objectives**: Characterization of individual retinal layer thicknesses in mild-to-moderate Parkinson’s disease (PD) remains limited. This study compares retinal layer thicknesses between patients with mild-to-moderate PD and controls. **Methods**: In this cross-sectional study, patients with PD of Hoehn and Yahr (HY) stage 1 to stage 3 and age- and sex-matched cognitively normal controls without PD underwent spectral-domain optical coherence tomography (OCT) imaging. Seven retinal layers were segmented: nerve fiber layer, ganglion cell–inner plexiform layer (GC-IPL), inner nuclear layer (INL), outer plexiform layer, outer nuclear layer (ONL), photoreceptor layer (PRL), and retinal pigment epithelium (RPE). Mean thickness for each layer and the total retina were measured within the 3 mm and 6 mm Early Treatment Diabetic Retinopathy Study rings. **Results**: Sixty-seven eyes from 41 patients with PD and 72 eyes from 41 controls were included. In the 6 mm ring, PD eyes had a nominally thicker GC-IPL (*p* = 0.01), INL (*p* = 0.01), RPE (*p* = 0.03), and total retina (*p* = 0.03) compared to control eyes. In the 6 mm ring, PRL was nominally thinner in HY stage 3 compared to HY stages 2–2.5 (*p* = 0.02). In the 6 mm ring, the ONL was nominally thinner in the eyes of PD patients in HY stage 3 compared to the eyes of PD patients in HY stages 1–1.5 (*p* = 0.02) and 2–2.5 (*p* = 0.04). However, none of these differences remained significant after False Discovery Rate adjustment (all adjusted *p* > 0.05). **Conclusions**: In this exploratory cross-sectional study, no significant differences in macular retinal layer thicknesses were found between patients with mild-to-moderate PD and controls without PD after adjusting for multiple comparisons. The nominal thickness differences observed here require larger, longitudinal cohorts to determine if true structural differences exist in mild-to-moderate PD.

## 1. Introduction

Parkinson’s disease (PD) is a progressive neurodegenerative disorder that affects 11.8 million people globally [1]. It is clinically defined by motor features such as resting tremor, bradykinesia, rigidity, and postural instability, though nonmotor features such as autonomic dysfunction, sleep disturbances, neuropsychiatric symptoms, and sensory abnormalities may be present [2,3]. Pathologically, PD is associated with degeneration of dopaminergic neurons in the substantia nigra pars compacta and intraneuronal accumulation of misfolded α-synuclein protein in Lewy bodies and Lewy neurites [4,5]. As an extension of the central nervous system, the retina often mirrors neurodegenerative processes occurring in the brain of patients with PD, including α-synuclein protein deposits [6], dopaminergic amacrine cell loss [7], and structural alterations [8].

Optical coherence tomography (OCT) has become a critical noninvasive tool for quantifying the microstructure of the retina with high resolution [9]. Previous research using OCT has established thinning of the inner retinal layers in patients with PD, specifically the macular nerve fiber layer (NFL) [10,11], ganglion cell–inner plexiform layer (GC-IPL) [12,13], and inner nuclear layer (INL) [13,14]. The photoreceptor layer (PRL) has also been shown to be thinner in patients with PD [15,16]. However, data regarding thickness changes in the other retinal layers such as the outer plexiform layer (OPL), outer nuclear layer (ONL), and retinal pigment epithelium (RPE) remain inconsistent. Studies have reported that in patients with PD, OPL thickness is greater [13,17] or unchanged [18,19,20]; ONL is thinner [13,21] or unchanged [18,19]; and RPE is thicker [19,22] or thinner [17,21] compared with controls. Furthermore, studies focusing on mild-to-moderate PD are lacking. Studying this particular population is important because disease-modifying therapies are most likely to succeed before severely symptomatic neurodegeneration occurs [23].

A more comprehensive understanding of structural changes in each retinal layer in mild-to-moderate PD could lead to the identification of noninvasive biomarkers. Such biomarkers have the potential to enable earlier disease detection, facilitate objective monitoring of disease progression, and serve as quantitative endpoints in future clinical trials for neuroprotective therapies [24,25]. In this study, we compared retinal layer thicknesses between mild-to-moderate PD patients and normal controls to identify possible OCT biomarkers in this population.

## 2. Materials and Methods

This was a cross-sectional, case–control study comparing retinal layer thicknesses in adult patients with mild-to-moderate PD to cognitively normal controls. This study was approved by the Duke Health Institutional Review Board (Pro00111831). All study activities adhered to the Declaration of Helsinki and the Health Insurance Portability and Accountability Act. Written informed consent was obtained prior to enrollment from all participants or their legally authorized representatives after explanation of the nature and possible consequences of the study.

### 2.1. Participant Eligibility and Recruitment

Patients with PD were recruited from the Duke Neurological Disorders Clinic. The diagnosis of PD was confirmed by fellowship-trained movement disorders neurology faculty using the United Kingdom Parkinson’s Disease Society Brain Bank Diagnostic Criteria, with each PD subject exhibiting bradykinesia plus at least one of the following: muscular rigidity, 4–6 Hz resting tremor, and/or postural instability [26]. Supporting their diagnosis, all PD patients taking levodopa had a beneficial response to the medication [27]. Demographic information, medical history, smoking history, Mini-Mental State Exam (MMSE) score, prescription eye drop use, and levodopa use at time of imaging were collected through patient interview or electronic health record review. To assess PD severity, patients were assigned a Hoehn and Yahr (HY) stage by Duke neurologists experienced in PD management. The PD cohort consisted of participants with HY stage 1–3 (consistent with mild-to-moderate disease), while patients found to have higher stages were excluded.

The control group consisted of individuals with no history of neurodegenerative disease or cognitive impairment. Controls were recruited from the surrounding community, the Duke Neurological Disorders Clinic, and the Duke Alzheimer’s Disease Prevention Registry. Controls were matched 1:1 to patients with PD by sex and age (±5 years).

Participants were excluded from the study if they had a comorbidity that could affect the retina and confound interpretation of whether retinal changes were due to PD. Exclusion criteria included a history of neurodegenerative disease other than PD, ocular surgery other than cataract or refractive surgery, diabetes, uncontrolled hypertension, glaucoma, optic neuropathy, vitreoretinal disorders (such as vitreomacular traction, epiretinal membranes, drusen, and reticular pseudodrusen), best corrected visual acuity worse than 20/40, or refractive error greater than +6 or less than −6 diopters. Participants were screened for these exclusion criteria at the time of enrollment via reported medical history, medical record review, and Snellen testing. Ultra-widefield scanning laser ophthalmoscopy (California, Optos, Marlborough, MA, USA) was carried out to further screen for vitreoretinal pathology.

### 2.2. Participant Characteristics

The study included 139 eyes from 82 participants: 67 eyes from 41 patients with PD and 72 eyes from 41 age- and sex-matched cognitively normal controls. The cohorts were well-matched in age (69.6 ± 8.6 years for patients with PD vs. 70.0 ± 7.6 years for controls) and sex (65.9% male, or 27/41, in both groups). Both groups were limited in terms of racial diversity (1 Black and the remaining White in both groups), and race data was missing in 2 control participants. The average MMSE score for patients with PD was significantly lower than that of controls (27.8 ± 3.0 vs. 29.4 ± 0.9; *p* < 0.001). Years of education was similar between groups (16.6 ± 2.5 years for PD vs. 16.8 ± 2.2 years for controls), though educational data was missing for a few participants (1 PD and 3 control participants). Additionally, similar proportions of participants in the PD and control groups had a previous smoking history (34.1% of PD patients and 34.3% of controls). Data on smoking history was missing for 6 control participants. Concurrent ocular medication use was rare: one control participant used latanoprost in one eye and timolol in the other eye due to glaucoma-suspect status, while one participant with PD used lifitegrast for dry eye disease. The average HY stage of PD patients was 2.0 ± 0.6. The mean PD duration was 6.3 ± 6.1 years. Almost all PD patients (87.8% or 36/41) had active prescriptions for levodopa at study enrollment (Table 1).

### 2.3. Imaging and Data Collection

To assess the thickness of retinal layers, trained study personnel conducted retinal imaging of undilated eyes using a spectral-domain OCT device (Cirrus HD-5000 AngioPlex, Carl Zeiss Meditec, Dublin, CA, USA). Simultaneous scanning laser ophthalmoscopy was used to confirm foveal centration. Trained study staff reviewed all images and excluded those with low signal strength (<7/10), poor foveal centration, or focal signal loss. High-quality 512 × 128 macular cube scans were imported to the Duke Reading Center’s DRCVisualizer software (version 2.1.1), and retinal layers were automatically segmented using a previously validated algorithm [28,29]. Seven retinal layers were segmented: NFL, GC-IPL, INL, OPL, ONL, PRL, and RPE.

Following automatic segmentation, research team members (MZ, ND, SDP) who had been trained by the Duke Reading Center manually reviewed each scan and corrected layer boundaries as needed, with additional input from a senior investigator (DSG) when necessary. Research team members were not blinded to diagnosis. Mean thickness measurements for each retinal layer and the total retinal thickness were then obtained for the 6 mm and 3 mm rings of the Early Treatment Diabetic Retinopathy Study (ETDRS) grid centered on the fovea, and results for both regions are reported in full. The 1 mm ETDRS circle was excluded from this analysis because the inner retinal layers are virtually absent at the foveola.

### 2.4. Statistical Analysis

All analyses were conducted using R (version 4.5.2). Demographic variables were analyzed using Fisher’s exact tests for categorical data and Wilcoxon rank-sum tests for continuous variables. Retinal layer thicknesses were compared between PD patients and controls using generalized estimating equations (GEE) to account for the correlation between eyes from the same individual. A second set of GEE was used to assess the relationship between retinal layer thicknesses and HY clusters. HY clusters were defined as follows: cluster 1 = HY stages 1–1.5; cluster 2 = HY stages 2–2.5; cluster 3 = HY stage 3. A third set of GEE was used to assess the relationship between retinal thicknesses and disease duration. GEE assumed a Gaussian distribution with an exchangeable correlation structure. The threshold for nominal statistical significance was set at a *p* < 0.05.

To control for multiple comparisons, *p*-values for retinal layer thickness comparisons were adjusted using the Benjamini–Hochberg False Discovery Rate (FDR), with statistical significance defined as an FDR-adjusted *p* < 0.05. Adjusted *p*-values for retinal layer thickness analyses were grouped as 3 families of tests: 16 tests for comparing eyes with PD to controls, 48 tests for HY cluster-based comparisons, and 16 tests for associations with disease duration.

To assess for observer bias, an independent research team member (A.H.), who was blinded to the participants’ clinical diagnoses, segmented a subset of 30 eyes, stratified to include representative numbers of control eyes and different stages of PD. Inter-rater reliability between the unblinded graders and the independent blinded grader was evaluated using the Intraclass Correlation Coefficient (ICC) for absolute agreement (two-way mixed-effects model, single rater) for both the 6 mm and 3 mm ETDRS rings.

## 3. Results

**Figure 1:**
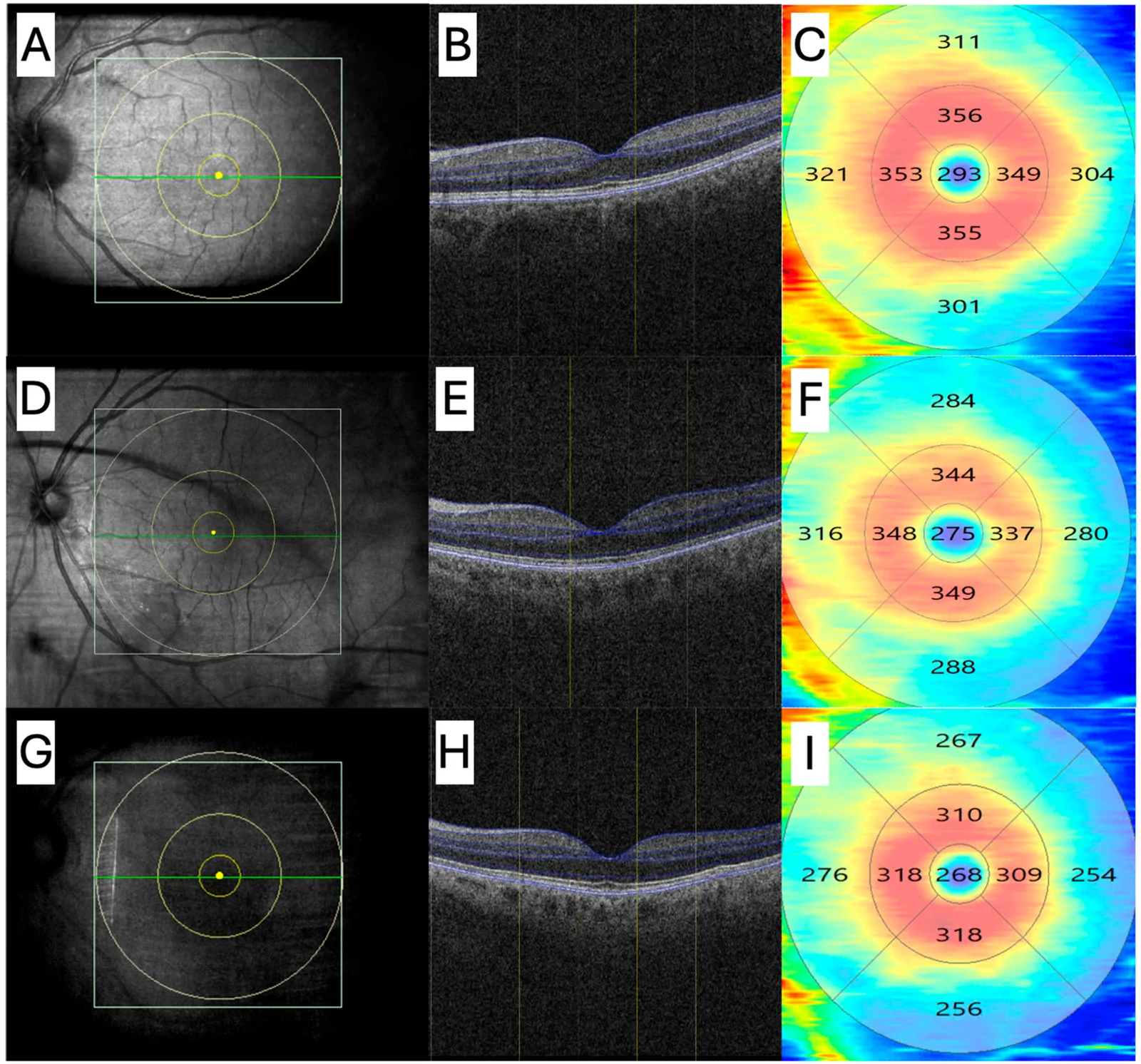
Representative scanning laser ophthalmoscopy (SLO) and macular optical coherence tomography scans from two patients with Parkinson’s disease (PD) and a cognitively normal control without PD. (**A**–**C**) Images from a 61-year-old female PD patient with Hoehn and Yahr (HY) stage 1. (**A**) SLO image with overlying Early Treatment Diabetic Retinopathy Study grid, yellow dot indicating the foveal center point, and green line corresponding to the B-scan; (**B**) segmented B-scan with blue lines indicating segmented boundaries between retinal layers; and (**C**) color-coded total retina thickness map. (**D**–**F**) Images from a 76-year-old female PD patient with HY stage 3. (**D**) SLO image, (**E**) segmented B-scan, and (**F**) color-coded total retina thickness map. (**G**–**I**) Images from a 66-year-old female cognitively normal control. (**G**) SLO image, (**H**) segmented B-scan, and (**I**) color-coded total retina thickness map.

The primary GEE analysis evaluated retinal layer thickness differences between PD eyes and control eyes in seven retinal layers and the total retina in the 6 mm and 3 mm ETDRS rings. In the 6 mm ring, PD eyes had a nominally thicker GC-IPL (61.06 ± 5.04 μm vs. 57.87 ± 5.47 μm, *p* = 0.01), INL (29.92 ± 2.20 μm vs. 28.44 ± 2.24 μm, *p* = 0.01), RPE (24.42 ± 2.64 μm vs. 23.23 ± 1.96 μm, *p* = 0.03), and total retina (288.30 ± 12.00 μm vs. 281.04 ± 13.31 μm, *p* = 0.03) compared to control eyes (representative images in Figure 1) (Table 2). No nominal differences in retinal layer thicknesses were found between PD eyes and control eyes in the 3 mm ring (all *p* > 0.05) (Table 3). No nominal differences survived FDR adjustment (all adjusted *p* > 0.05).

The secondary GEE analyses evaluated differences in retinal layer thicknesses in patients of different PD stages and disease durations. In the 6 mm ring, the ONL was nominally thinner in eyes of patients in HY stage 3 compared to eyes of patients in HY stages 1–1.5 (54.14 ± 3.30 μm vs. 58.57 ± 5.92 μm, *p* = 0.02) and HY stages 2–2.5 (54.14 ± 3.30 μm vs. 57.23 ± 5.25 μm, *p* = 0.04). In the 6 mm ring, the PRL was nominally thinner in eyes of patients in HY stage 3 compared to eyes of patients in HY stages 2–2.5 (48.73 ± 1.98 μm vs. 51.78 ± 3.58 μm, *p* = 0.02) (Supplementary Table S1). No nominal differences in retinal layer thicknesses were found between PD eyes of different stages in the 3 mm ring (all *p* > 0.05) (Supplementary Table S2). No nominal relationships were found between retinal layer thicknesses and disease duration (Supplementary Tables S3 and S4). No nominal differences survived FDR adjustment (all *p* > 0.05).

Inter-rater reliability was excellent across the inner and middle retinal layers in both rings, including the total retina (ICC = 0.999 for 6 mm; 0.997 for 3 mm), NFL (ICC = 0.989 for 6 mm; 0.974 for 3 mm), GC-IPL (ICC = 0.999 for 6 mm; 0.996 for 3 mm), INL (ICC = 0.999 for 6 mm; 0.995 for 3 mm), OPL (ICC = 0.996 for 6 mm; 0.997 for 3 mm), and ONL (ICC = 0.999 for both 6 mm and 3 mm). However, reliability was poor for the PRL (ICC = 0.239 for 6 mm; 0.208 for 3 mm) and the RPE (ICC = −0.037 for 6 mm; 0.001 for 3 mm).

A post hoc power analysis was conducted for GC-IPL thickness in the 6 mm ring. To account for inter-eye clustering, an effective sample size was calculated using the observed inter-eye correlation (ρ = 0.955) and a design effect of 1.66. Adjusting the alpha threshold to 0.003 to approximate multiple comparison corrections across 16 primary tests, this study achieved 39.8% statistical power to detect the observed effect size (Cohen’s d = 0.61).

## 4. Discussion

This cross-sectional study compared retinal layer thicknesses between mild-to-moderate PD and control participants. The INL, GC-IPL, RPE, and total retina were nominally thicker in our cohort of PD patients compared to controls, though these differences did not survive correction for multiple comparisons. Previous studies have generally reported that PD is associated with atrophic thinning of inner retinal layers and the PRL [10,11,12,13,14,15,16]. Our findings raise the possibility that subtle thickening of specific retinal layers is associated with mild-to-moderate PD. These structural changes warrant further investigation to determine whether they truly exist and whether they have value as biomarkers to detect or track the progression of PD.

Compared to control subjects, patients with mild-to-moderate PD had nominally thicker INL and GC-IPL. This contrasts with prior research which generally shows that inner retinal layers, including GC-IPL and INL, are thinner in PD patients compared to controls [12,13,14]. However, those studies have typically enrolled patients with more advanced PD, including average HY stages of 3.9 ± 1.1 and 2.3 ± 1.0. Given that our cohort had PD with an average HY stage of 2.0 ± 0.6, our trend toward thickening may represent a distinct phase of PD-related retinal changes, though this hypothesis remains speculative. Notably, one of the few studies showing GC-IPL thickening specifically enrolled patients with PD with duration of less than 5 years; patients from that study had an average HY stage of 1.8 ± 0.6 [30]. It is possible that neuroinflammation physically expands the inner retina in mild PD, in a process analogous to the early thickening found in the retina of patients with mild cognitive impairment, a precursor to Alzheimer’s disease [31].

We also observed a nominally thicker RPE in patients with PD compared to controls. The literature on RPE thickness is inconsistent, showing variable increase or decrease for PD patients compared to controls [17,19,21,22]. Studies showing RPE thinning in PD varied greatly in average HY stage of their cohorts (2.3 ± 1.0, 1.2 ± 0.3, and 2.9 ± 0.4), and the studies showing RPE thickening did not report HY stages. Several mechanisms have been proposed to explain retinal thickening in PD, including inflammatory and metabolic changes [19,32,33]. However, the biological significance of these nominal differences remains uncertain and should be interpreted cautiously given poor inter-rater reliability for the PRL-RPE boundary.

In our secondary analyses, GC-IPL, INL, and RPE thicknesses did not significantly vary with disease duration or differ by HY stage. If a distinct structural response occurs in mild-to-moderate PD, its temporal relationship with disease progression remains unclear. Alternatively, rather than occurring in all patients with PD, subtle inner retinal thickening may reflect a specific phenotype or be associated with clinical features not captured by the HY scale. The mechanism and validity of these nominal differences require further longitudinal investigation.

In our cohort, the PRL was nominally thinner in the eyes of PD patients in HY stage 3 compared to the eyes of PD patients in HY stages 2–2.5. Additionally, the ONL was nominally thinner in the eyes of PD patients in HY stage 3 compared to the eyes of PD patients in HY stages 1–1.5 and 2–2.5. This is consistent with some previous research showing that PD is associated with thinning of the PRL [15,16] and ONL [13,21], though other research contradicts these findings [18,19]. The cause of these findings is unclear. While these findings suggest that PRL and ONL thinning may track with motor symptom severity, we did not find any association between retinal layer thicknesses and overall disease duration.

The difference in findings between the 6 mm and 3 mm ETDRS rings may suggest spatial specificity of PD-related retinal changes. Nominal differences between PD patients and controls were isolated to the 6 mm ring. Since the 3 mm ETDRS ring is anatomically thicker, small disease-related differences may be less apparent there, whereas the thinner 6 mm ring may be more sensitive to subtle structural changes.

Importantly, the absolute differences in retinal layer thickness between the cohorts were smaller than the corresponding within-group standard deviations, indicating substantial overlap between retinal layer thicknesses in PD and control eyes. The same observation applied to differences in retinal layer thicknesses between HY clusters. Their reproducibility across cohorts, devices, and segmentation methods remains to be established. Even if these micrometer-level differences are confirmed in larger cohorts, normal physiological variation and the non-specific nature of retinal layer thickness changes limit their diagnostic utility when used in isolation. Combining OCT parameters with other imaging and serological biomarkers and clinical assessment may improve discrimination between groups compared with any modality alone [23]. Whether longitudinal OCT measurements can detect within-patient structural change or track PD progression requires prospective study.

This study has several limitations. The sample size was modest (67 eyes from PD patients, 72 eyes from control participants), which may have limited our ability to detect small but meaningful differences. Additionally, an exploratory post hoc power calculation, using an effective sample size to approximate inter-eye correlation and an alpha threshold of 0.003 to account for multiple testing, estimated approximately 40% power to detect the observed GC-IPL effect. This reinforces the exploratory nature of the findings and underscores the necessity of larger sample sizes in future studies. More PD eyes were excluded than controls (15 PD eyes vs. 10 control eyes), which is largely due to the difficulty of positioning symptomatic PD patients for imaging. Our reliability analysis revealed that while other retinal layer segmentations were highly reproducible across graders, the boundary separating the PRL and RPE exhibited poor absolute agreement in both the 6 mm and 3 mm rings. Consequently, our findings in the PRL and RPE should be interpreted with caution until segmentation algorithms for their boundary are further refined. Findings were nominally significant and did not survive correction for multiple comparisons using the Benjamini–Hochberg procedure; therefore, they should be interpreted as exploratory. Axial length measurements were unavailable, so magnification correction was not performed. Although eyes with refractive error outside ± 6 diopters were excluded, residual differences in axial length could have influenced the measured layer thicknesses, potentially confounding thickness measurements. Our study cohort lacked racial diversity, limiting the generalizability of our findings. Finally, misclassification of cases was possible as PD diagnoses were made clinically by expert neurologists but not confirmed pathologically.

The cause of the observed nominal retinal thickening in mild-to-moderate PD remains unclear. Assessing microvascular metrics could help differentiate whether thickening is driven by neuroinflammation-induced vasodilation or ischemia-related vessel loss. Future studies should incorporate OCT angiography to evaluate retinal vascular structure. Since almost all PD patients were taking levodopa at the time of enrollment, the relationship between levodopa prescription and retinal layer thickness could not be assessed. Without a drug-naive PD subgroup analysis, the confounding effect of levodopa cannot be ruled out, which restricts the generalizability of these findings. Furthermore, this study lacks corresponding molecular and functional data. Therefore, it is unclear how these structural trends correlate with visual function or molecular mechanisms. Future prospective studies should integrate visual testing and fluid biomarkers to better understand the pathology and clinical impact of these retinal changes.

This cross-sectional study found no significant differences in retinal layer thicknesses between patients with mild-to-moderate PD and controls after correction for multiple comparisons. However, mild-to-moderate PD was associated with nominal thickening of the INL, GC-IPL, RPE, and total retina. HY stage 3 was associated with nominal thinning of PRL and ONL compared to milder stages of PD. Further research is necessary to explore the usefulness of OCT in detecting true structural changes in PD and to determine whether these structural changes have utility for diagnosis, serving as endpoints in clinical trials, or monitoring disease progression.

## Figures and Tables

**Table 1:** Participant characteristics.

Variable	Statistic	Control	PD	*p*-Value
Age, years	*N*	41	41	0.95
	Mean (SD)	70.0 (7.6)	69.6 (8.6)	
	Min, Median, Max	52.7, 71.1, 83.1	48.1, 70.8, 86.8	
Male sex	*n*/*N* (%)	27/41 (65.9)	27/41 (65.9)	1.00
Race	*n*/*N* (%)			1.00
White		38/39 (97.4)	40/41 (97.6)	
Black		1/39 (2.6)	1/41 (2.4)	
Mini-Mental State Exam	*N*	41	41	<0.001
	Mean (SD)	29.4 (0.9)	27.8 (3.0)	
	Min, Median, Max	26, 30, 30	14, 29, 30	
Years of education	*N*	38	40	0.70
	Mean (SD)	16.8 (2.2)	16.6 (2.5)	
	Min, Median, Max	12, 16, 22	12, 17, 21	
Smoking History	*n*/*N* (%)	12/35 (34.3)	14/41 (34.1)	1.00
Hoehn and Yahr stage	*n*/*N* (%)			
1			6/41 (14.6)	
1.5			3/41 (7.3)	
2			21/41 (51.2)	
2.5			7/41 (17.1)	
3			4/41 (9.8)	
Disease duration, years	*N*		41	
	Mean (SD)		6.3 (6.1)	
	Min, Median, Max		0.2, 4.3, 26.6	
Levodopa prescription	*n*/*N* (%)		36/41 (87.8)	

*p*-values for continuous variables were calculated with Wilcoxon rank-sum tests. *p*-values for categorical variables were calculated with Fisher’s exact tests. N = number of participants. PD = Parkinson’s disease. SD = standard deviation. Min = minimum. Max = maximum.

**Table 2:** Generalized estimating equations results comparing retinal layer thicknesses of Parkinson’s disease patients vs. controls in 6 mm ETDRS ring.

Retinal Layer	Statistic	Control	PD	*p*-Value	FDR *p*-Value
Total retina, µm	*N*	72	67	0.03	0.12
	Mean (SD)	281.04 (13.31)	288.30 (12.00)		
	Min, Median, Max	251.98, 281.81, 316.91	244.65, 287.41, 321.79		
Nerve fiber layer, µm	*N*	72	67	0.25	0.39
	Mean (SD)	38.56 (5.09)	39.67 (4.43)		
	Min, Median, Max	27.52, 37.91, 49.03	31.12, 39.31, 53.74		
Ganglion cell–inner plexiform layer, µm	*N*	72	67	0.01	0.11
	Mean (SD)	57.87 (5.47)	61.06 (5.04)		
	Min, Median, Max	45.19, 57.18, 68.40	43.92, 60.13, 72.68		
Inner nuclear layer, µm	*N*	72	67	0.01	0.11
	Mean (SD)	28.44 (2.24)	29.92 (2.20)		
	Min, Median, Max	24.81, 28.60, 34.28	25.01, 30.21, 34.29		
Outer plexiform layer, µm	*N*	72	67	0.91	0.91
	Mean (SD)	24.78 (1.69)	24.76 (1.53)		
	Min, Median, Max	21.94, 24.47, 29.63	22.12, 24.62, 29.26		
Outer nuclear layer, µm	*N*	72	67	0.40	0.53
	Mean (SD)	55.82 (5.77)	57.30 (5.33)		
	Min, Median, Max	44.04, 55.90, 69.84	42.20, 57.45, 69.65		
Photoreceptor layer, µm	*N*	72	67	0.07	0.23
	Mean (SD)	52.35 (2.88)	51.18 (3.49)		
	Min, Median, Max	46.40, 52.31, 59.86	45.21, 50.70, 59.62		
Retinal pigment epithelium, µm	*N*	72	67	0.03	0.12
	Mean (SD)	23.23 (1.96)	24.42 (2.64)		
	Min, Median, Max	20.03, 22.90, 28.75	18.64, 24.50, 30.42		

*p*-values were based on Z-test of difference between group means using generalized estimating equations to account for the correlations between eyes of the same patient. False Discovery Rate (FDR)-corrected *p*-values were calculated using the Benjamini–Hochberg procedure across all PD vs. control models (16 tests). N = number of eyes. PD = Parkinson’s disease. ETDRS = Early Treatment Diabetic Retinopathy Study. SD = standard deviation. Min = minimum. Max = maximum.

**Table 3:** Generalized estimating equations results comparing retinal layer thicknesses of Parkinson’s disease patients vs. controls in 3 mm ETDRS ring.

Retinal Layer	Statistic	Control	PD	*p*-Value	FDR *p*-Value
Total retina, µm	*N*	72	67	0.13	0.25
	Mean (SD)	328.92 (15.83)	334.93 (14.69)		
	Min, Median, Max	293.90, 326.44, 374.58	297.39, 336.41, 372.30		
Nerve fiber layer, µm	*N*	72	67	0.24	0.39
	Mean (SD)	25.72 (2.10)	26.25 (2.11)		
	Min, Median, Max	22.32, 25.20, 33.65	23.32, 26.10, 33.27		
Ganglion cell–inner plexiform layer, µm	*N*	72	67	0.10	0.23
	Mean (SD)	86.57 (8.81)	89.79 (7.64)		
	Min, Median, Max	70.75, 86.86, 104.50	73.24, 89.07, 109.96		
Inner nuclear layer, µm	*N*	72	67	0.09	0.23
	Mean (SD)	37.80 (2.19)	38.79 (2.15)		
	Min, Median, Max	32.90, 37.65, 44.06	33.48, 39.06, 42.69		
Outer plexiform layer, µm	*N*	72	67	0.31	0.45
	Mean (SD)	30.18 (3.73)	29.36 (3.19)		
	Min, Median, Max	24.64, 29.30, 39.39	24.94, 28.74, 38.85		
Outer nuclear layer, µm	*N*	72	67	0.43	0.53
	Mean (SD)	70.56 (8.01)	72.43 (7.46)		
	Min, Median, Max	53.90, 71.26, 85.67	51.18, 73.85, 85.66		
Photoreceptor layer, µm	*N*	72	67	0.76	0.82
	Mean (SD)	50.97 (3.65)	50.80 (4.45)		
	Min, Median, Max	44.10, 50.19, 61.53	43.04, 50.28, 61.39		
Retinal pigment epithelium, µm	*N*	72	67	0.53	0.61
	Mean (SD)	27.11 (2.78)	27.52 (3.49)		
	Min, Median, Max	20.96, 26.54, 32.55	17.57, 27.89, 32.71		

*p*-values are based on Z-test of difference between group means using generalized estimating equations to account for the correlations between eyes of the same patient. False Discovery Rate (FDR)-corrected *p*-values were calculated using the Benjamini–Hochberg procedure across all PD vs. control models (16 tests). N = number of eyes. PD = Parkinson’s disease. ETDRS = Early Treatment Diabetic Retinopathy Study. SD = standard deviation. Min = minimum. Max = maximum.

## Data Availability

The datasets generated and/or analyzed during the current study are not publicly available due to patient confidentiality but may be available from the corresponding author on reasonable request.

## Supplementary Materials

The following supporting information can be downloaded at https://www.mdpi.com/article/10.3390/diagnostics16203412/s1, Table S1: Generalized Estimating Equations Results Showing Retinal Layer Thicknesses in Parkinson’s Disease Patients by HY Cluster in 6 mm ETDRS Ring; Table S2: Generalized Estimating Equations Results Showing Retinal Layer Thicknesses in Parkinson’s Disease Patients by HY Cluster in 3 mm ETDRS Ring; Table S3: Generalized Estimating Equations Results Showing Retinal Layer Thicknesses vs. Disease Duration in Parkinson’s Disease Patients in 6 mm ETDRS Ring; Table S4: Generalized Estimating Equations Results Showing Retinal Layer Thicknesses vs. Disease Duration in Parkinson’s Disease Patients in 3 mm ETDRS Ring.

## Abbreviations

The following abbreviations are used in this manuscript:

ETDRS	Early Treatment Diabetic Retinopathy Study
FDR	False Discovery Rate
GC-IPL	Ganglion Cell–Inner Plexiform Layer
GEE	Generalized Estimating Equations
HY	Hoehn and Yahr
ICC	Intraclass Correlation Coefficient
INL	Inner Nuclear Layer
Max	Maximum
Min	Minimum
MMSE	Mini-Mental State Exam
NFL	Nerve Fiber Layer
OCT	Optical Coherence Tomography
ONL	Outer Nuclear Layer
OPL	Outer Plexiform Layer
PD	Parkinson’s Disease
PRL	Photoreceptor Layer
RPE	Retinal Pigment Epithelium
SD	Standard Deviation
SLO	Scanning Laser Ophthalmoscopy
