# Supplementary material for: Macular Retinal Layer Thickness in Mild-to-Moderate Parkinson’s Disease
Source: Diagnostics (Basel). 2026 Oct 20;16(20):3412. doi: 10.3390/diagnostics16203412 (PMC13703910; doi:10.3390/diagnostics16203412)
Supplement: Supplementary file 1 [file diagnostics-16-03412-s001.zip › diagnostics-4506058-supplementary.pdf]

Table S1. Generalized Estimating Equations Results Showing Retinal Layer Thicknesses in Parkinson's Disease Patients by HY Cluster in 6-mm ETDRS Ring.

Retinal Layer	Statistic	HY Cluster 1	HY Cluster 2	HY Cluster 3	Cluster 1 vs. 2 P-value (FDR)	Cluster 1 vs. 3 P-value (FDR)	Cluster 2 vs. 3 P-value (FDR)
**Total retina, µm**	N	15	47	5	0.26 (0.84)	0.25 (0.84)	0.81 (0.98)
	Mean (SD)	293.04 (16.04)	287.15 (10.60)	284.87 (8.33)			
	Min, Median, Max	272.99, 287.41, 321.79	244.65, 288.65, 310.33	271.28, 285.47, 292.02			
**Nerve fiber layer, µm**	N	15	47	5	0.08 (0.65)	0.78 (0.98)	0.23 (0.84)
	Mean (SD)	41.76 (4.91)	38.76 (3.75)	41.99 (6.66)			
	Min, Median, Max	34.50, 43.35, 48.33	31.12, 38.15, 47.86	37.74, 39.31, 53.74			
**Ganglion cell-inner plexiform layer, µm**	N	15	47	5	0.44 (0.91)	0.75 (0.98)	0.77 (0.98)
	Mean (SD)	62.69 (6.96)	60.56 (4.23)	60.86 (5.51)			
	Min, Median, Max	52.70, 64.73, 72.68	43.92, 60.13, 67.68	55.83, 58.97, 70.28			
**Inner nuclear layer, µm**	N	15	47	5	0.92 (0.98)	0.37 (0.84)	0.08 (0.65)
	Mean (SD)	30.04 (3.08)	30.02 (1.94)	28.65 (1.19)			
	Min, Median, Max	25.85, 30.50, 34.29	25.01, 30.23, 33.56	27.52, 28.46, 30.64			
**Outer plexiform layer, µm**	N	15	47	5	0.49 (0.98)	0.34 (0.84)	0.53 (0.98)
	Mean (SD)	24.52 (1.54)	24.81 (1.52)	24.97 (1.90)			
	Min, Median, Max	22.27, 24.51, 28.53	22.12, 24.62, 29.26	22.79, 24.85, 27.75			
**Outer nuclear layer, µm**	N	15	47	5	0.35 (0.84)	**0.02** (0.51)	**0.04** (0.65)
	Mean (SD)	58.57 (5.92)	57.23 (5.25)	54.14 (3.30)			
	Min, Median, Max	50.02, 60.17, 69.65	42.20, 57.45, 68.44	50.52, 52.64, 58.04			
**Photoreceptor layer, µm**	N	15	47	5	0.33 (0.84)	0.33 (0.84)	**0.02** (0.51)
	Mean (SD)	50.12 (3.09)	51.78 (3.58)	48.73 (1.98)			
	Min, Median, Max	46.30, 49.65, 54.91	45.21, 51.31, 59.62	46.18, 48.73, 51.72			
**Retinal pigment epithelium, µm**	N	15	47	5	0.08 (0.65)	0.71 (0.98)	0.75 (0.98)
	Mean (SD)	25.34 (1.83)	24.00 (2.62)	25.53 (4.25)			
	Min, Median, Max	22.02, 25.21, 28.94	18.64, 23.99, 30.02	19.45, 25.19, 30.42			

P-values were based on Z-test of difference between group means using generalized estimating equations to account for the correlations between eyes of the same patient. False Discovery Rate (FDR) corrected p-values were calculated using the Benjamini-Hochberg procedure across all retinal layer thickness vs. HY cluster models (48 tests). Hoehn and Yahr (HY) clusters were defined as follows: cluster 1 = HY stages 1-1.5; cluster 2 = HY stages 2-2.5; cluster 3 = HY stage 3. N = number of eyes. ETDRS = Early Treatment Diabetic Retinopathy Study. SD = standard deviation. Min = minimum. Max = maximum.

Table S2. Generalized Estimating Equations Results Showing Retinal Layer Thicknesses in Parkinson's Disease Patients by HY Cluster in 3-mm ETDRS Ring.

Retinal Layer	Statistic	HY Cluster 1	HY Cluster 2	HY Cluster 3	Cluster 1 vs. 2 P-value (FDR)	Cluster 1 vs. 3 P-value (FDR)	Cluster 2 vs. 3 P-value (FDR)
**Total retina, µm**	N	15	47	5	0.92 (0.98)	0.98 (0.99)	0.86 (0.98)
	Mean (SD)	334.37 (19.94)	335.42 (13.27)	332.09 (11.25)			
	Min, Median, Max	301.83, 336.41, 372.30	297.39, 337.17, 370.61	319.94, 331.37, 344.47			
**Nerve fiber layer, µm**	N	15	47	5	0.29 (0.84)	0.88 (0.98)	0.65 (0.98)
	Mean (SD)	26.81 (2.41)	26.08 (1.96)	26.22 (2.71)			
	Min, Median, Max	23.46, 26.78, 32.67	23.32, 25.93, 33.27	23.93, 26.24, 30.61			
**Ganglion cell-inner plexiform layer, µm**	N	15	47	5	0.73 (0.98)	0.99 (0.99)	0.81 (0.98)
	Mean (SD)	90.57 (8.04)	89.74 (7.36)	87.96 (10.43)			
	Min, Median, Max	75.59, 92.36, 104.85	73.24, 89.07, 109.96	76.36, 88.52, 100.95			
**Inner nuclear layer, µm**	N	15	47	5	0.40 (0.87)	0.67 (0.98)	0.28 (0.84)
	Mean (SD)	38.31 (2.49)	39.03 (1.99)	38.01 (2.53)			
	Min, Median, Max	34.23, 38.01, 42.69	33.48, 39.06, 42.21	34.41, 39.20, 40.20			
**Outer plexiform layer, µm**	N	15	47	5	0.24 (0.84)	0.11 (0.75)	0.21 (0.84)
	Mean (SD)	28.58 (2.38)	29.37 (3.02)	31.59 (5.92)			
	Min, Median, Max	26.07, 28.56, 34.60	24.94, 28.84, 37.77	25.47, 32.02, 38.85			
**Outer nuclear layer, µm**	N	15	47	5	0.72 (0.98)	0.31 (0.84)	0.34 (0.84)
	Mean (SD)	72.61 (9.24)	72.60 (6.95)	70.22 (7.64)			
	Min, Median, Max	51.18, 74.42, 85.66	53.92, 73.32, 83.08	61.74, 71.37, 78.12			
**Photoreceptor layer, µm**	N	15	47	5	0.88 (0.98)	0.97 (0.99)	0.91 (0.98)
	Mean (SD)	50.43 (4.84)	51.04 (4.47)	49.66 (3.45)			
	Min, Median, Max	45.48, 49.26, 60.68	43.04, 50.61, 61.39	45.44, 50.47, 53.11			
**Retinal pigment epithelium, µm**	N	15	47	5	0.79 (0.98)	0.73 (0.98)	0.83 (0.98)
	Mean (SD)	27.06 (4.08)	27.56 (3.33)	28.44 (3.65)			
	Min, Median, Max	18.77, 27.84, 31.71	17.57, 27.85, 32.71	22.13, 30.14, 31.15			

P-values were based on Z-test of difference between group means using generalized estimating equations to account for the correlations between eyes of the same patient. False Discovery Rate (FDR) corrected p-values were calculated using the Benjamini-Hochberg procedure across all retinal layer thickness vs. HY cluster models (48 tests). Hoehn and Yahr (HY) clusters were defined as follows: cluster 1 = HY stages 1-1.5; cluster 2 = HY stages 2-2.5; cluster 3 = HY stage 3. N = number of eyes. ETDRS = Early Treatment Diabetic Retinopathy Study. SD = standard deviation. Min = minimum. Max = maximum.

Table S3. Generalized Estimating Equations Results Showing Retinal Layer Thicknesses vs. Disease Duration in Parkinson's Disease Patients in 6-mm ETDRS Ring.

Retinal Layer	Coefficient (SE)	95% CI	P-value	FDR P-value
**Total retina, µm**	-0.31 (0.24)	[-0.78, 0.17]	0.20	0.33
**Nerve fiber layer, µm**	-0.19 (0.10)	[-0.39, 0.01]	0.06	0.32
**Ganglion cell-inner plexiform layer, µm**	0.03 (0.15)	[-0.26, 0.33]	0.83	0.83
**Inner nuclear layer, µm**	-0.08 (0.06)	[-0.19, 0.03]	0.16	0.32
**Outer plexiform layer, µm**	0.02 (0.04)	[-0.06, 0.10]	0.64	0.69
**Outer nuclear layer, µm**	-0.08 (0.09)	[-0.25, 0.09]	0.34	0.45
**Photoreceptor layer, µm**	-0.10 (0.07)	[-0.23, 0.03]	0.14	0.32
**Retinal pigment epithelium, µm**	0.08 (0.06)	[-0.03, 0.19]	0.14	0.32

P-values were based on generalized estimating equations to account for the correlations between eyes of the same patient, evaluating disease duration (years) as a continuous predictor for retinal layer thickness. False Discovery Rate (FDR) corrected p-values were calculated using the Benjamini-Hochberg procedure across all retinal layer thickness vs. disease duration models (16 tests). ETDRS = Early Treatment Diabetic Retinopathy Study. SE = standard error. CI = confidence interval.

Table S4. Generalized Estimating Equations Results Showing Retinal Layer Thicknesses vs. Disease Duration in Parkinson's Disease Patients in 3-mm ETDRS Ring.

Retinal Layer	Coefficient (SE)	95% CI	P-value	FDR P-value
**Total retina, µm**	-0.44 (0.35)	[-1.13, 0.24]	0.21	0.33
**Nerve fiber layer, µm**	-0.07 (0.05)	[-0.17, 0.02]	0.14	0.32
**Ganglion cell-inner plexiform layer, µm**	-0.13 (0.24)	[-0.61, 0.34]	0.58	0.67
**Inner nuclear layer, µm**	-0.11 (0.06)	[-0.23, 0.01]	0.06	0.32
**Outer plexiform layer, µm**	0.06 (0.12)	[-0.16, 0.29]	0.58	0.67
**Outer nuclear layer, µm**	-0.21 (0.12)	[-0.46, 0.03]	0.09	0.32
**Photoreceptor layer, µm**	-0.09 (0.07)	[-0.23, 0.06]	0.25	0.36
**Retinal pigment epithelium, µm**	0.09 (0.05)	[-0.01, 0.19]	0.09	0.32

P-values were based on generalized estimating equations to account for the correlations between eyes of the same patient, evaluating disease duration (years) as a continuous predictor for retinal layer thickness. False Discovery Rate (FDR) corrected p-values were calculated using the Benjamini-Hochberg procedure across all retinal layer thickness vs. disease duration models (16 tests). ETDRS = Early Treatment Diabetic Retinopathy Study. SE = standard error. CI = confidence interval.
